# Characterization of a *Propionibacterium acnes* Surface Protein as a Fibrinogen-Binding Protein

**DOI:** 10.1038/s41598-017-06940-3

**Published:** 2017-07-25

**Authors:** Philippe A. Grange, Joël Raingeaud, Willy Morelle, Anne-Geneviève Marcelin, Vincent Calvez, Nicolas Dupin

**Affiliations:** 1 Université Sorbonne Paris Descartes, Faculté de Médecine, INSERM CNRS UMR8104, Institut Cochin U1016, Laboratoire de Dermatologie-CNR Syphilis, Paris, France; 2 INSERM U981, Institut Gustave Roussy, Villejuif, France; 3 UMR CNRS/USTL 8576, Unité de Glycobiologie Structurale et Fonctionnelle, Université des Science et Technologies de Lille 1, Villeneuve-d’Ascq, France; 4 AP-HP, Groupe Hospitalier Pitié-Salpêtrière – Charles Foix, Service de Virologie – INSERM 1136-UMR UPMC Paris 6, Paris, France; 5 AP-HP, Groupe Hospitalier Paris Centre Cochin-Hôtel Dieu-Broca, Service de Dermatologie-Vénéréologie, Paris, France

## Abstract

*Propionibacterium acnes* (*P. acnes*) is a major skin-associated bacterium that was long considered commensal, until several studies revealed it to be an opportunistic pathogen. We investigated the ability of *P. acnes* surface proteins to recognize ECM proteins and showed that a 58 kDa *P. acnes* surface protein was specifically recognized by human fibrinogen (hFg). The 58 kDa protein was further characterized by two-dimensional (2-D) electrophoresis and MALDI-ToF as a *P. acnes* host cell-surface attachment protein, PA25957, recognizing dermatan sulfate (DsA1). This protein sequence contains 432 amino acids with the presence of three structurally different domains: an N-terminal signal peptide, a C-terminal LPXTG motif, and a PT repeat region. DsA1 is mostly produced during stationary phase. It appears to be highly glycosylated, containing GalNAc residues. Purified DsA1 strongly recognizes the Aα and Bβ subunits of hFg, and specific enzymatic deglycosylation of hFg demonstrated the involvement of the protein backbone in the recognition process. The Bβ subunit of hFg was cloned in four peptide fractions (Fg1-Fg4). The N-terminal Fg1 peptide of hFg was recognized by DsA1, and priming DsA1 with Fg1 inhibited DsA1/hFg recognition. We describe here for the first time, the characterization of a *P. acnes* surface glycoprotein recognizing human fibrinogen.

## Introduction

The principal cutaneous commensal species in the genus *Propionibacterium* are *P. acnes*, *P. granulosum*, *P. lymphophilum*, *P. propionicum* and *P. avidum*. *P. acnes*, previously known as *Corynebacterium parvum*, is a gram-positive, aerotolerant-anaerobic non-sporulating bacterium that has been described as diphtheroid or coryneform. Further studies distinguished these species from other gram-positive bacteria on the basis of its cell wall and outer envelope. It contains phosphatidylinositol and a peptidoglycan with a region of peptide chains including L,L-diaminopimelic acid and D-alanine^1^.

*P*. acnes belongs to the normal skin microbiota and was long considered to be commensal, until several studies revealed that it was actually an opportunistic pathogen involved in several invasive infections^2^. Indeed, *P. acnes* is commonly isolated from inflammatory acne on skin^3^, but it has also been found in cases of late-stage prosthetic joint infections, endocarditis, endophthalmitis, osteomyelitis, and shunt-associated central nervous system infections^4–8^. It is also suspected to play a role in the etiology of sarcoidosis^9^, and in prostate cancer, in which it is thought to contribute to the induction of continuous low-grade inflammation and the downregulation of cell cycle progression^10^. *P. acnes* can form biofilms and is often isolated from specimens obtained from patients with biofilm infections of medical implants^11^.

The genome of *P. acnes* is 2.5 Mb in size and has been completely sequenced^12^. Based on the presence of galactosyl residues on the bacterial surface, *P. acnes* strains were initially classified into two serotypes, I and II. Further genetic analysis identified another phylotype, type III, and subdivisions within the type I clade (IA_1_, IA_2_, IB, IC) based on the ability of strains to cause inflammation, the production of putative virulence factors, resistance to antibiotic treatment and ability to colonize different areas of the host^13–16^. The discovery of these differences between *P. acnes* phylotypes led to the proposal that *P. acnes* should be divided into subspecies, with type III as *P. acnes* subsp. *elongatum*, type I as *P. acnes* subsp. *acnes* and type II as *P. acnes* subsp. *defendens* ^17, 18^. Recent whole-genome analyses and studies of peptidoglycan composition led to the suggestion that three new genera should be recognized as *Acidipropionibacterium*, *Pseudopropionibacterium* and *Cutibacterium*, with *P. acnes* becoming *C. acnes* ^19^.

*P. acnes* has genes encoding metabolic enzymes enabling it to survive in microaerophilic conditions, but also lipases that degrade the lipids of the pilosebaceous follicle, providing the bacterium with the energy it needs. *P. acnes* also has genes encoding putative surface proteins containing the LPXTG anchor sequence potentially involved in the activation of innate immunity and adhesion^12, 20^. *P. acnes* can stimulate the production of interleukins IL-1α/β, IL-8, IL-12, TNF-α, and β-defensins by keratinocytes, sebocytes and monocytes *in vitro* and *in vivo*, in acne lesions. The production of these molecules is triggered by stimulation of the TLR2 receptor and activation of the NF-κB and MAPK signaling pathways, and via the NLRP3 inflammasome pathway. In addition, *P. acnes* CAMP factor 1 protein, which recognizes TLR2 on the surface of keratinocytes, induces the production of large amounts of CXCL8^21^. *P. acnes* can also induce the massive production of reactive oxygen species (ROS) by keratinocytes, helping to initiate the inflammatory reaction^22–26^.

In many pathogenic bacteria, the invasion of host cells is mediated by bacterial surface proteins or adhesins specifically recognizing extracellular matrix (ECM) components, ideal microbial adhesion targets used by many pathogens for tissue colonization and the initiation of infection. Skin-associated bacteria, such as those of the genera *Staphylococcus* and *Streptococcus*, express numerous cell surface adhesins called MSCRAMMs (microbial surface components recognizing adhesive matrix molecules), which specifically bind to host ECM components to promote bacterial adhesion to target cells, and subsequently, to initiate colonization and infection^27^. *P. acnes* can adhere to human skin^28^ but can also cause deeper infections by traveling from the surface to the infection site through nonspecific interactions and then undergoing an irreversible adhesion process through specific binding, as described elsewhere^29^. *P. acnes* has been shown to bind to human epithelial cells^30^. However, despite extensive investigations of the inflammatory reaction caused by *P. acnes*, little is known about the surface protein of *P. acnes* putatively involved in the recognition of ECM components. In this study, we investigated the interaction of *P. acnes* surface proteins with ECM components. By using a Western blot ligand-binding assays we identified a 58 kDa glycoprotein specifically recognized by human fibrinogen, involving the N-terminal part of the protein backbone of the fibrinogen.

## Results

### *P. acnes* surface proteins bind to eukaryotic ligands

**Figure 1:**
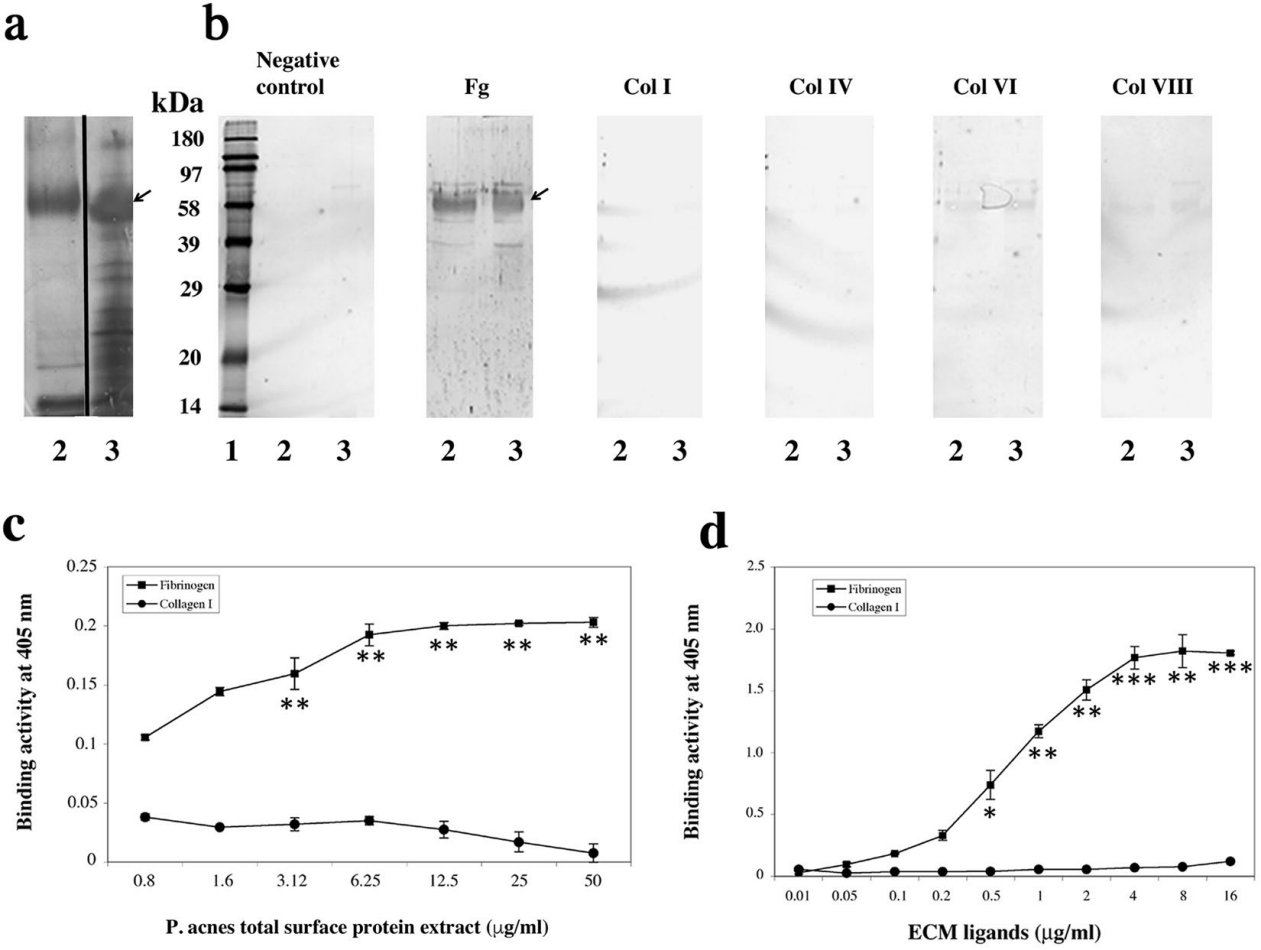
Identification of a 58 kDa *P. acnes* surface protein recognized by human fibrinogen. (**a**) Proteins were separated by 12.5% SDS-PAGE (10 μg per lane) and detected by silver staining. (**b**) Proteins (50 μg per lane) were incubated with biotinylated human fibrinogen, collagens I, IV, VI and VIII (Fg, ColI, ColIV, Col VI, Col VIII; 0.1 μg/ml) and detected with HRP-streptavidin. Control experiments were performed with HRP-streptavidin alone. Lane 1: molecular weight standards. Lanes 2 and 3: surface proteins extracted at 60 °C in PBS and at 45 °C in 1 M LiCl, respectively. The arrow indicates the position of the 58 kDa protein. (**c**) Immobilized proteins (0.8 to 50 μg/ml) were probed with biotinylated fibrinogen (0.5 μg/ml). (**d**) Immobilized proteins (25 μg/ml) were probed with biotinylated fibrinogen (0.1 to 16 μg/ml). Data are presented as the mean ± standard deviation of three independent experiments. Statistical significance is indicated by *P ≤ 0.05, **P ≤ 0.01, ***P ≤ 0.001.

*P. acnes* surface proteins were extracted by heating the bacterial suspension in the presence or absence of lithium chloride. *P. acnes* total protein extracts were subjected to electrophoresis and the bands were detected by silver staining of the gel (Fig. 1a). Several bands ranging from 14 to 100 kDa in size were detected, but a single protein with an apparent molecular mass of 58 kDa accounted for >90% of total proteins (Fig. 1a). Interestingly, more proteins appeared to be extracted in the presence of lithium chloride than in its absence (Fig. 1a, lanes 2 and 3). Putative *P. acnes* surface adhesins were identified by Western blotting with biotinylated ligands (Fig. 1b). The 58 kDa protein was recognized by biotinylated hFg in both extracts (Fig. 1b, lanes 2 and 3), and another protein band was detected at about 39 kDa. No recognition was detected with collagens I and IV (Col I, Col IV), and recognition was very faint and non-reproducible, and was therefore considered nonspecific, with collagens VI and VIII (Col VI, Col VIII). For confirmation of these results, several concentrations of concentrated surface protein were immobilized onto polysterene plates and probed with biotinylated hFg and human collagen I (Fig. 1c). Fibrinogen-binding activity was detected, reaching a plateau at about 6.25 μg/ml protein, but no binding activity was detected with collagen I (Fig. 1c). We checked these findings, by immobilizing concentrated surface protein extract (25 μg/ml) on the plates and incubating them with various amounts of biotinylated hFg and hCollagen I (Fig. 1d). Strong, dose-dependent and saturable binding was observed with hFg, suggesting a possible saturation of recognized sites. No binding activity was detected with collagen I, indicating that the interaction between hFg and the *P. acnes* surface proteins was specific. These results are consistent with the qualitative results previously reported. As recognition of the 58 kDa protein by hFg appeared to be specific, we characterized this protein further.

### Characterization of the 58 kDa protein

**Figure 2:**
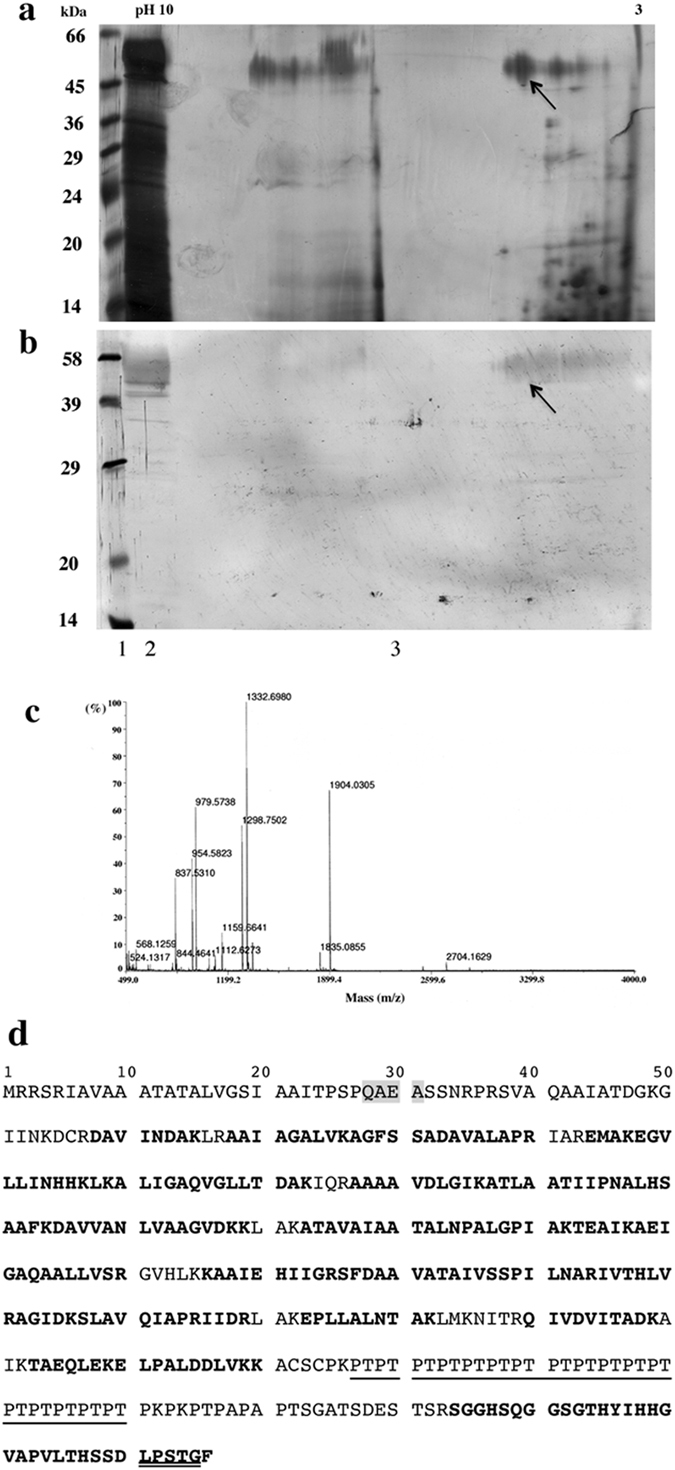
Characterization of the 58 kDa *P. acnes* surface protein. Concentrated proteins (200 μg) were separated by 2D-electrophoresis. (**a**) Proteins were detected by silver staining. (**b**) Fibrinogen binding activity was assessed with biotinylated human fibrinogen (0.1 μg/ml). Lane 1: molecular weight standard. Lane 2: sample separated by 10% SDS-PAGE (1DE) only (50 μg of protein). Lane 3: sample after 2DE. The arrow indicates the spot excised for identification by MALDI-ToF. (**c**) MALDI-ToF spectrum obtained for the spot of interest. (**d**) Peptide sequence of the 58 kDa protein with 64% coverage (in bold). The sequence signal is highlighted in gray, the repeated PT domain is underlined, and the LPXTG motif is double-underlined. Based on its binding capacities (to hFg, this study; and to dermatan sulfate^31, 32^), the 58 kDa protein was abbreviated DsA1.

Concentrated surface protein extract was subjected to 2-D gel electrophoresis. The first dimension was IEF over a broad pI range, extending from 10 to 3. The second dimension was SDS-PAGE in a 12.5% acrylamide gel, with protein detection by silver staining (Fig. 2a). We observed about 50 protein spots after separation (Fig. 2a). The 58 kDa protein spot was localized by running a second gel in parallel, for Western ligand blotting with biotinylated hFg (Fig. 2b). The 58 kDa protein corresponded to two major spots (Fig. 2b, lane 3) matching the spots detected on the silver-stained gel (Fig. 2a, lane 3). The protein spot of interest was excised and characterized by MALDI-ToF on peptide mixtures after in-gel digestion. Figure 2c shows the MALDI mass spectrum of a tryptic peptide mixture produced from the protein spot arrowed in Fig. 2a. Twenty-two experimentally obtained tryptic peptide masses were found to match predicted peptide masses to within 0.1 Da, covering 64% of the amino-acid sequence. Protein sequence database searches identified the protein as a *P. acnes* host cell-surface attachment protein (Table 1) (GenBank accession number DQ469873.1, locus tag PA25957), DsA1, also known to recognize dermatan-sulfate^31, 32^. The PA25957 gene product is a 432-amino acid protein, with a predicted molecular mass of about 44 kDa, a signal peptide sequence (QAEA) at position 28, 17 tandem repeats of a proline- and threonine-rich region at the C terminus, from positions 327 to 360 (Fig. 2d), and a LPXTG motif in position 411, at the C-terminus, corresponding to a cell-anchoring motif and possible sortase site, consistent with a surface location. The 58 kDa fibrinogen-binding protein was further abbreviated with DsA1.

**Table 1:** Measured and calculated molecular masses for tryptic peptides.

Position	Mass (Da)^b^				Sequence
Start-End	Observed	Measured	Calculated	Difference	
58–65	844.4641	843.4568	844.4290	−0.9722	R.DAVINDAK.L
68–76	813.5099	812.5026	812.5120	−0.0093	R.AAIAGALVK.A
77–90	1332.6980	1331.6907	1331.6834	0.0074	K.AGFSSADAVALAPR.I
77–90	1333.1610	1332.1537	1331.6834	0.4704	K.AGFSSADAVALAPR.I
94–107	1618.7115	1617.7042	1617.8661	−0.1618	R.EMAKEGVLLINHHK.L
98–107	1159.6641	1158.6568	1158.6509	0.0059	K.EGVLLINHHK.L
108–123	1609.9697	1608.9624	1609.9767	−1.0142	K.LKALIGAQVGLLTDAK.I
110–123	1369.8074	1368.8001	1368.7977	0.0025	K.ALIGAQVGLLTDAK.I
127–136	928.5557	927.5484	927.5389	0.0095	R.AAAAVDLGIK.A
137–154	1810.0007	1808.9934	1809.0148	−0.0214	K.ATLAATIIPNALHSAAFK.D
155–168	1341.7325	1340.7252	1340.7300	−0.0048	K.DAVVANLVAAGVDK.K
155–169	1469.8439	1468.8366	1468.8249	0.0117	K.DAVVANLVAAGVDKK.L
173–192	1834.0825	1833.0752	1833.0723	0.0029	K.ATAVAIAATALNPALGPIAK.T
193–210	1841.0198	1840.0125	1840.0418	−0.0292	K.TEAIKAEIGAQAALLVSR.G
198–21	1297.7482	1296.7409	1297.7354	−0.9944	K.AEIGAQAALLVSR.G
198–210	1298.7502	1297.7429	1297.7354	0.0076	K.AEIGAQAALLVSR.G
198–210	1299.1985	1298.1912	1297.7354	0.4559	K.AEIGAQAALLVSR.G
216–225	1107.6747	1106.6674	1106.6560	0.0114	K.KAAIEHIIGR.S
217–225	979.5738	978.5665	978.5610	0.0055	K.AAIEHIIGR.S
217–225	979.9589	978.9516	978.5610	0.3906	K.AAIEHIIGR.S
226–244	1903.0289	1902.0216	1902.0211	0.0006	R.SFDAAVATAIVSSPILNAR.I
226–244	1903.5977	1902.5904	1902.0211	0.5694	R.SFDAAVATAIVSSPILNAR.I
245–251	837.5310	836.5237	836.5232	0.0005	R.IVTHLVR.A
252–256	503.2774	502.2701	502.2751	−0.0050	R.AGIDK.S
257–265	954.5823	953.5750	953.5658	0.0092	K.SLAVQIAPR.I
257–265	954.9483	953.9410	953.5658	0.3752	K.SLAVQIAPR.I
266–269	516.3015	515.2942	515.3067	−0.0125	R.IIDR.L
273–282	1068.5443	1067.5370	1068.6179	−1.0809	K.EPLLALNTAK.L
273–282	1069.6302	1068.6229	1068.6179	0.0050	K.EPLLALNTAK.L
290–299	1101.6213	1100.6140	1100.6077	0.0063	R.QIVDVITADK.A
303–309	818.4270	817.4197	817.4181	0.0016	K.TAEQLEK.E
310–319	1112.6273	1111.6200	1111.6125	0.0076	K.ELPALDDLVK.K
310–320	1239.7582	1238.7509	1239.7074	−0.9565	K.ELPALDDLVKK.A
384–416	3284.4987	3283.4914	3283.5396	−0.0481	R.SGGHSQGGSGTHYIHHGVAPVLTHSSDLPSTGF.–
384–416	3285.3900	3284.3827	3283.5396	0.8432	R.SGGHSQGGSGTHYIHHGVAPVLTHSSDLPSTGF.–

^a^These peptides, which identified the 58 kDa protein, correspond to 267 of the 416 residues, corresponding to 64% sequence coverage (See also Fig. 2). ^b^Mono-isotopic masses.

### DsA1 is mostly expressed during stationary phase

**Figure 3:**
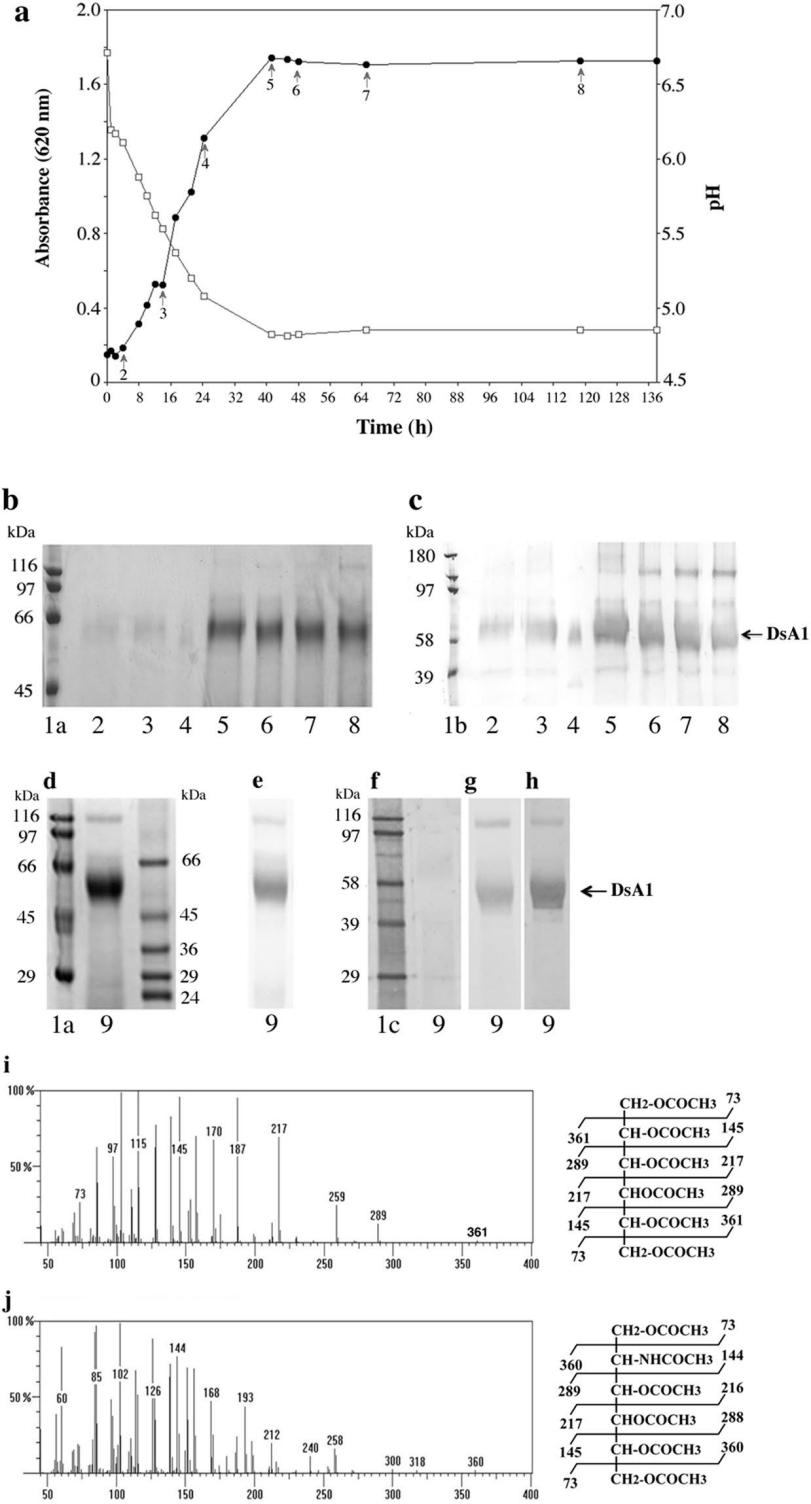
DsA1 characteristics. (**a**) Growth curve and pH variation over the culture period. (**b**) Protein (50 μg/lane) were separated by 10% SDS-PAGE and detected by Coomassie blue staining. (**c**) Binding of surface proteins to biotinylated hFg (0.1 μg/ml). Lanes 1a and 1b correspond to unlabeled and biotinylated molecular weight standards, respectively. Lanes 2–8: fractions (2 to 8) arrowed on the growth curve in panel a. Concentrated surface protein (Lane 9, 50 μg) was subjected to 10% SDS-PAGE and stained with (**d**) Coomassie blue, (**e**) PAS. Binding assays were then carried out with (**f**) HRP-streptavidin alone, or with plant lectins (**g**) DBA and (**h**) SBA (0.2 μg/ml). Plant lectins ConA, PNA, SNA, MAL II, RCA I, UEA I, WGA (see Table 2 for their specificities) were also tested but no recognition was detected. Molecular weight standards were used unlabeled (Lanes 1a and 1b), and biotinylated (Lane 1c). Electron impact mass spectrum of the peaks eluted on the GC chromatogram at the positions of (**i**) galactose, glucose and (**j**) N-acetyl-galactosamine. The mass spectra for galactose and N-acetyl-galactosamine are shown (the mass spectra of galactose and glucose are identical).

For studies of the expression kinetics of DsA1, two-liter cultures of *P. acnes* were grown under anaerobic conditions, at 37 °C, with stirring. Aliquots were taken from the culture medium at various times points, over a period of 140 h. Absorbance and pH were measured on 3 ml aliquots, bacteria were recovered by centrifugation and the surface proteins were extracted by heating in the presence of LiCl. *P. acnes* is a slow-growing bacterium, with an exponential growth phase extending over a period of about 30 h (Fig. 3a). DsA1 was barely detectable during the exponential phase of culture, but was strongly detected in 40-hours cultures, by SDS-PAGE and Western blotting with biotinylated hFg (Fig. 3b,c; lane 5), with a decrease in intensity after 120 h of growth (Fig. 3c, lane 8). Interestingly, a second protein of about 150 kDa was recognized by hFg; this protein appeared after 48 h growth, during the stationary phase (Fig. 3c, lanes 6–8).

### DsA1 is glycosylated

The surface proteins of prokaryotic organisms can be glycosylated^33^, and the reported differences between the apparent and theoretical molecular masses of this protein suggests that they may bear carbohydrates. We analyzed the presence of carbohydrate residues on DsA1, by subjecting concentrated surface proteins to electrophoresis (Fig. 3d), transferring the bands to PVDF membranes and staining with periodic acid-Schiff reagent (PAS) (Fig. 3e). The high intensity of PAS staining observed suggests that DsA1 contains large amounts of carbohydrates. For preliminary characterization of the carbohydrates linked to the protein backbone, we used biotinylated plant lectins known to recognize carbohydrates in a specific manner (Table 2). DsA1 was recognized by the plant lectins SBA and DBA only (Fig. 3g,h), consistent with the presence of GalNAc residues on the protein. No recognition was found with the HRP alone (Fig. 3f). For confirmation of these results, DsA1 was subjected to electrophoresis and the resulting bands were transferred to PVDF membrane. The carbohydrate components were then analyzed by gas chromatography-mass spectrometry (GC-MS). Alditol acetates prepared from the PVDF-bound protein band reveaeds the presence of mannose, galactose, glucose and GalNAc residues (Fig. 3i,j).

**Table 2:** Plant lectins used in this study.

Plant lectins and their abbreviations		Sugar specificity
Concanavalin A	ConA	α-Man or less α -Glc
Soybean agglutinin	SBA	α/β GalNAc* and less Gal
Peanut agglutinin	PNA	Gal β(1–3) GalNAc (-NeuAc)
*Sambucus nigra*	SNA	NeuAc α(2–6)
*Maackia amurensis*	MAL II	NeuAc α(2–3)
*Ricinus communis*	RCA I	β Gal* and less GalNAc
Jacalin		Gal β(1–3) GalNAc or T Ag (on O-glycoconjugates)
*Ulex europaeus*	UEA I	α Fuc (on proteins and lipids)
*Dolichos biflorus*	DBA	α GalNAc
Wheatgerm	WGA	GlcNAc

*In a terminal position.

### DsA1 binding specificity

**Figure 4:**
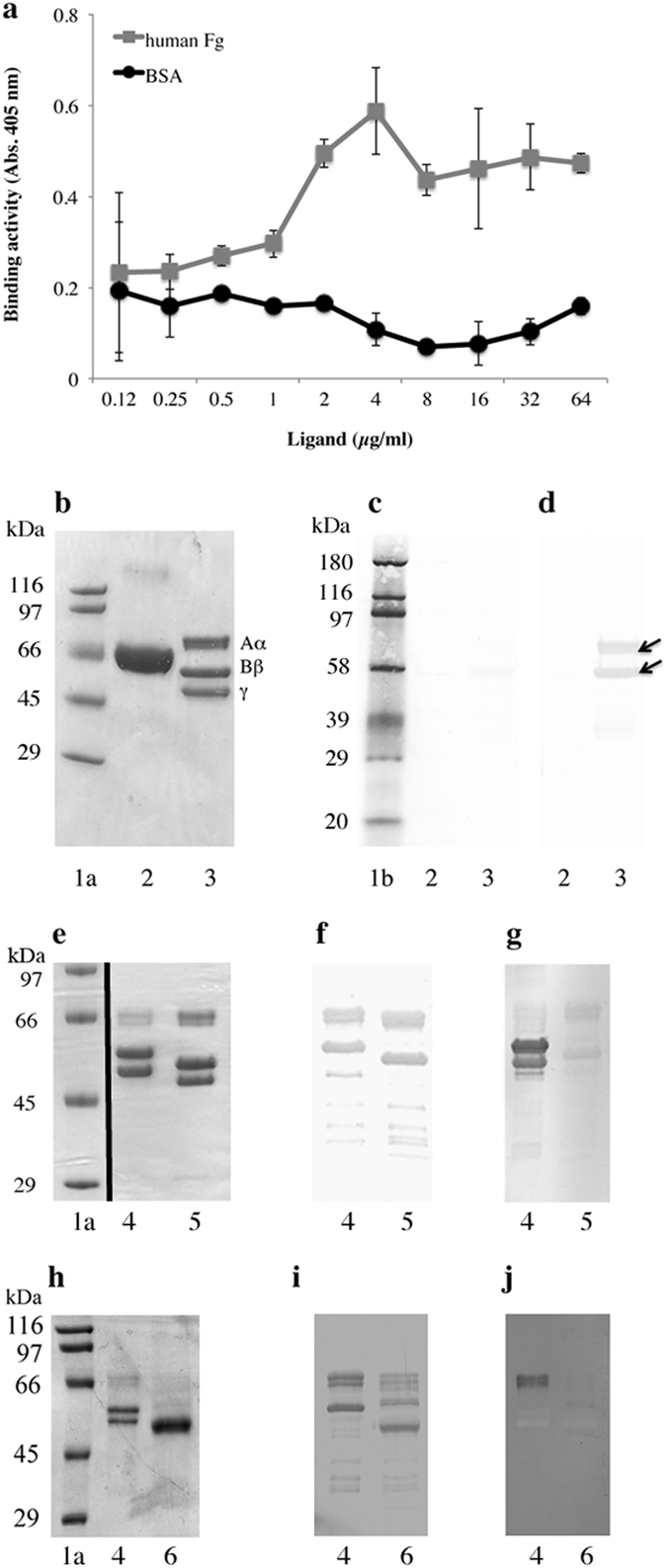
Binding of purified DsA1 to fibrinogen. (**a**) Various quantities of immobilized hFg were incubated with biotinylated DsA1 (0.1 μg/ml). Bovine serum albumin (BSA) was used as a negative control. (**b**) Commassie blue staining of proteins (10 μg per lane) separated by 10% SDS-PAGE. (**c**) HRP- streptavidin detection alone. (**d**) DsA1 binding (0.1 μg/ml). Purified hFg was treated with (**e,f,g**) N-glycosidase F (PNGAse F) and (**h,i,j**) O-glycosidase. (**e,h**) Treated and untreated hFg (10 μg per lane) were separated by 10% SDS-PAGE and detected by Coomassie blue staining. (**f,i**) Binding activity with biotinylated DsA1 (0.1 μg/ml). Biotinylated (**g**) RCA-I and (**j**) jacalin lectins binding activities were used as a deglycosylation control, respectively. Lanes 1a and 1b correspond to unlabeled and biotinylated molecular weight standards, respectively. Lanes 2 and 3: BSA and hFg, respectively. Lanes 4 and 5: Untreated and enzymaticaly treated hFg, respectively.

To assess the binding specificity, DsA1 was purified and results of a typical purification run are summarized in Supplementary Table S1. Salt precipitation of a large volume of *P. acnes* lithium chloride extract resulted in a small increase in the specific activity (1.45-fold increase in hFg-binding activity per unit of protein) of the concentrated extract (Supplementary Table S1 and Supplementary Fig. S1a,b). Concentrated surface protein extract was fractionated on an anion exchange column (Supplementary Fig. S1c), which removed a large amount of contaminating protein, from a concentration of 180 mM NaCl. Fractions with hFg-binding activity were eluted at NaCl concentrations starting from 160 mM (Supplementary Fig. S1c). This fraction contained a large proportion of DsA1, together with small amounts of contaminating proteins (Supplementary Fig. S1a,b). The final purification step of DsA1 was achieved by Sephacryl high-resolution gel filtration (Supplementary Fig. S1d), which removed all the protein contaminants (Supplementary Fig. S1a,b). In total, 0.23 mg of pure DsA1 with a specific activity of 1840 U/mg was obtained after this step (Supplementary Table S1). Purified DsA1 was biotinylated and used to analyze the nature of its interaction with hFg. Human Fg was immobilized on polysterene plates and dose-dependent binding activity was observed with biotinylated DsA1, whereas bovine serum albumin, used as a negative control, was not recognized (Fig. 4a). We then performed Western blot binding assays with DsA1 and separated hFg (Fig. 4b,c,d), which showed that only the Aα and Bβ subunits of hFg were recognized (Fig. 4d, lane 3). Neither the γ subunit nor the serum albumin used as control was recognized (Fig. 4d, lane 2). These results are consistent with those obtained for immobilized hFg and demonstrate the specificity of the recognition between DsA1 and hFg. It has been shown that hFg is a glycoprotein containing both N- and O-linked glycans^34, 35^. We investigated the part of the glycoprotein involved in recognition by DsA1, by treating hFg with PNGase F and O-glycosidase, for the specific removal of N- and O-linked glycans, respectively, from the protein backbone. The control of deglycosylation was assessed by electrophoretic mobililty shift analysis and by assessing RCA-I plant lectin binding (Fig. 4e,g, lanes 4 and 5). Removal of the N-linked glycans had no effect on DsA1 recognition (Fig. 4f, lane 5). Similar results were obtained after the removal of O-linked glycans (Fig. 4i, lane 6), for which deglycosylation controls shown that enzymatic treatment induced a protein electrophoretic mobility shift (Fig. 4h, lane 6) and abolished recognition by the plant lectin jacalin (Fig. 4j, lane 6). Thus, the protein backbone of hFg is involved in recognition by DsA1.

### An N-terminal peptide derived from fibrinogen inhibits the interaction between DsA1 and Fg

**Figure 5:**
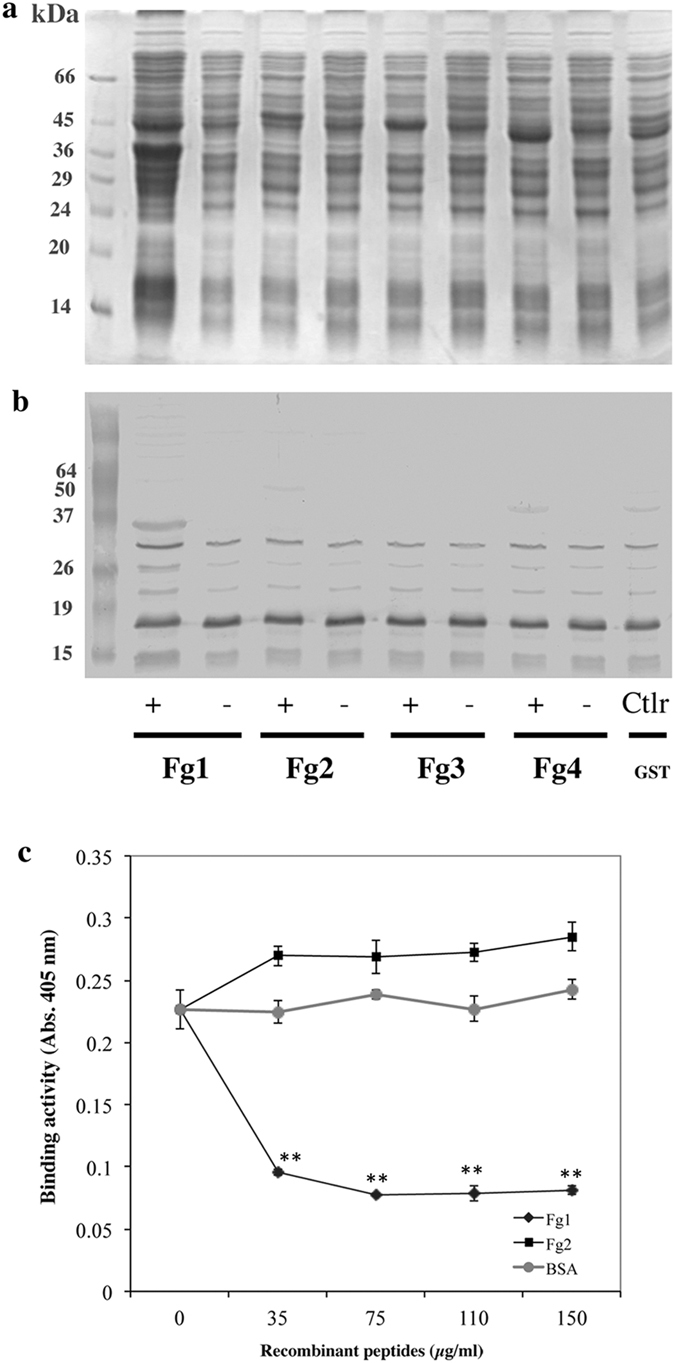
Inhibition of DsA1 binding to fibrinogen by a peptide derived from the N-terminus of fibrinogen. (**a**) GST-recombinant proteins (Fg1, Fg2, Fg3, Fg4) were expressed in *E. coli* (see Supplementary Figs S1 and S2), fractionated by 12.5% SDS-PAGE and detected by Coomassie blue staining. (**b**) Proteins were probed with biotinylated DsA1 (0.1 μg/ml). (**c**) Binding activity to hFg (25 μg) with pretreated biotinylated DsA1 with purified recombinant peptides Fg1 (♦), Fg2 (■) or BSA as control (●). Data are presented as the mean ± standard deviation of four independent experiments. Statistical significance is indicated by *P ≤ 0.05, **P ≤ 0.01.

The Bβ subunit of hFg was arbitrarily split into four sequences of equal length (Fg1, Fg2, Fg3, Fg4), which were tested for recognition by DsA1. The amplicons, containing the restriction sites for *Eco*RI and *Xho*I, were purified and inserted into the pBluescript SK + plasmid, for production of the Fgs inserts, which were then inserted into the pGEX-4F-2 expression plasmid (Supplementary Figs S2 and S3). Recombinant clones in *E. coli* clones were subjected to IPTG induction, and proteins produced were then analyzed by electrophoresis. Recombinant proteins with an apparent molecular mass of 37 kDa for Fg1 and 43 kDa for Fg2, Fg3, and Fg4, were detected after induction (Fig. 5a), and incubation with biotinylated DsA1 led to the recognition of Fg1 only (Fig. 5b). The Fg1 recombinant peptide was purified and incubated with DsA1, to test its ability to recognize immobilized hFg. Purified recombinant Fg2 peptide and BSA were used as negative controls. The Fg1 peptide strongly decreased the recognition of hFg by DsA1, whereas the Fg2 peptide and BSA did not (Fig. 5c).

## Discussion

Many skin-associated bacteria, such as *Staphylococcus aureus and Streptococcus pyogenes*, express molecules (proteins, lipoproteins) on their surface that can interact with proteins present in the extracellular matrix (ECM). These bacterial surface molecules are known as MSCRAMMs (microbial surface components recognizing adhesive matrix molecules) and have been implicated in virulence^36^. The surface proteins of *P. acnes* potentially involved in such recognition remain unknown. However, complete sequencing of the *P. acnes* genome has revealed the presence of genes encoding proteins with properties typical of surface proteins, which could potentially interact with host proteins^12^. The ECM of mammalian tissues is a stable three-dimensional macromolecular structure that underlies epithelial and endothelial cells. It consists of structural glycoproteins, such as collagen, laminin, fibronectin, and fibrinogen^37^, and the surface proteins of *P. acnes* have been shown to include an 80 kDa protein capable of recognizing fibronectin^38^. We obtained a *P. acnes* surface protein extract by heating in the presence and absence of high concentrations of lithium chloride, for the specific removal of surface proteins^21^, which were then resolved by electrophoresis. Western ligand blots experiments with several biotinylated ECM proteins identified a 58 kDa *P. acnes* surface protein that specifically recognized fibrinogen, but not collagens I, IV, VI and VIII. Yields were higher for the extraction of surface proteins in the presence of lithium chloride, as shown by the larger amount of the 58 kDa protein recognized by hFg, consistent with a surface location for the extracted proteins and a greater efficacy of lithium chloride for stripping surface components. Fibrinogen recognition was confirmed by quantifying the binding of *P. acnes* surface proteins to soluble hFg and human collagen I immobilized on plates. No recognition was detected with collagen I, whereas hFg was recognized in a saturable, dose-dependent manner. We then used a proteomic approach to characterize the 58 kDa protein, which was identified as the host cell-surface attachment protein PA25957^31^. This protein has an LPXTG cell-wall anchoring motif at its C-terminus, as also described for *S. aureus* surface proteins recognizing hFg (clumping factors ClfA and ClfB)^39^, providing further evidence for a surface location of this protein. However, PA25957 has a similar structure to the M-like protein of *S. equi*, despite low levels of sequence similarity between these two proteins^31^. The 58 kDa protein sequence also contains a signal peptide cleavage site (QAEA) and a PTRP (proline-threonine repeat protein) sequence repeat, consistent with the ability of the 58 kDa protein to recognize hFg, because cell wall-associated proteins with tandem repeats have been shown to be associated with the binding domains of other proteins or polysaccharides^40^. However, further investigations will be required to determine whether the PTRP motif of the 58 kDa protein is responsible for hFg-binding activity. The PA25957 protein has been shown to bind dermatan sulfate, and has thus been called DsA1 (for dermatan sulfate-binding adhesin 1)^32^. It has also been shown to have different molecular masses in different strains, due to variation in the number of PT repeats^31^. The PA25957 protein is one of the top four most antigenic proteins of *P. acnes* and may be considered to be a MSCRAMM (microbial surface component recognizing adhesive matrix molecules)^31, 32^. PA25957/DsA1 has been reported to display no affinity for collagen I, consistent with our results. As PA25957 was found to bind both dermatan sulfate and hFg, we used the abbreviation DsA1.

DsA1 was produced principally during stationary phase, consistent with the in-clone phase/antigenic variation of DsA1 expression which has been previously observed by IFM analysis with a monoclonal antibody (QUBPa1) to the protein DsA1^31^. PA25957/DsA1 was found to be secreted in the supernantant of *P. acnes* strain 266 at mid-exponential growth phase^20, 41^. Here, we analyzed the presence of DsA1 in washed bacterial pellets from a *P. acnes* strain grown on a medium recommended for the isolation of anaerobic organisms. This difference in the medium used may account for discrepancies between the results of different studies. Moreover, the *S. aureus* hFg-binding Eap (extracellular adhesion protein) was described as a secreted protein present in substantial amounts in the culture supernatant. However, hFg-binding Eap does not seem to be covalently anchored to the bacterial surface and has no LPXTG motif, consistent with its secretion^42^. Interestingly, PA25597/DsA1 has an LPXTG motif but neither sortase substrate (membrane-spanning domain and charged carboxy-tail) and it is thought that PA25957/DsA1 may be both associated with the cell surface and secreted, as some strains have no LPXTG motif^31^. Further studies will be required to analyze DsA1 expression on a large number of *P. acnes* strains of all the different phylotypes, and to determine how this surface-anchored protein is secreted by bacteria. During the stationary phase we also detected the presence of a 150 kDa protein recognized by hFg. We cannot exclude that this Fg-binding protein could be an aggregate of DsA1. However, it should be noted that electrophoretic analysis were performed under denaturizing conditions including a reducing agent. More investigations will be necessary to determine the relationship between the 58- and the 150-kDa hFg-binding proteins.

A difference was found between the apparent molecular mass of DsA1 (about 58 kDa) estimated by SDS-PAGE and its predicted molecular mass of 44 kDa. This discrepancy was also found in a previous study^32^, and high proline content was put forward as a possible explanation^31^. However, the presence of carbohydrate residues on the protein backbone may also contribute to the higher apparent molecular mass. Preliminary carbohydrate analysis strongly suggested that DsA1 was a glycoprotein containing N-acetylgalactosaminyl (GalNAc) residues, as shown by PAS staining, recognition by plant lectins that specifically recognize GalNAc residues, and GC-MS analysis. Bacteria have been shown to express several glycan-based compounds involved in their physiology on their surfaces^43^. In particular, glycoproteins have been characterized as the cell wall-anchored SRR adhesin for platelets in *S. aureus* containing N-acetylglucosaminyl (GlcNAc) residues^44^. *P. acnes* phylotypes I and II were initially distinguished on the basis of the absence of galactosyl residues and differences in lipoglycan composition in type II^45^. These differences suggested that *P. acnes* might possess a glycosyltransferase system capable of synthesizing glycan-based structures, a hypothesis confirmed by the genome sequence of *P. acnes*, which contains several genes encoding glycosyltransferases^12^. Further studies on large amounts of purified protein will be required for full characterization of the structure of the glycan moiety of DsA1 and its role in hFg binding.

Fibrinogen is a 340 kDa plasma glycoprotein precursor of fibrin, which is involved in platelet aggregation. Each fibrinogen molecule consists of three pairs of non-identical polypeptide chains (Aα_2_Bβ_2_γ_2_) arranged such that all six N-termini are located in the central part of the molecule. The Aα-chains consist of 610 residues and have a molecular mass of 67 kDa, the Bβ-chain is a 55 kDa polypeptide composed of 461 residues, and the 48 kDa γ-chain has 411 residues^46^. We assessed the nature of the binding between DsA1 and hFg, by purifying DsA1 and incubating it with immobilized, and electrophoretically separated hFg. DsA1 was able to recognize only the hFg and more precisely the Aα and Bβ subunits after electrophoretic separation of hFg. These results suggest that recognition is specific and involves only the protein part of the molecule, as shown by the removal of glycans from hFg. We focused on the Bβ chain of hFg and showed that only the N-terminal peptide was recognized by DsA1, and that use of the N-terminal Fg1 peptide inhibited the recognition between DsA1 and whole hFg. These results strongly suggest that the interaction between hFg and DsA1 involves the N-terminal part of hFg.

It has been shown previously that *P. acnes* strains isolated from acne lesions are different from strains isolated from healthy skin^47^. More particularly, the type IA_1_ was preferentially found in strains from acne while the types IA_2_, IC, II and III were more commonly found in healthy individuals or associated with opportunistic infections such as endocarditis, osteomyelitis, and post-surgical infections after the implantation of prosthetic material^45, 48–50^. The 58 kDa hFg-binding protein identified here was obtained from *P. acnes* strain 6919 (NCTC737), consistent with previous findings that DsA1 is produced by *P. acnes* types IA_1_, IA_2_ and IC, but not in types IB, II and III based on slipped strand mispairing which lead to introduction of stop codons in the DsA1 sequence^13, 31, 32, 41, 51^. As previously proposed, it would suggest that other factors may be implicated in the progress of the physiopathology of acne^13^. Some factors could be specifically produced by bacteria and have yet to be characterized and/or the balance between acne- and health-associated species and metagenomics elements of the skin microbiota would determine the formation of acne or health^52^. Since the expression of DsA1 appears to be restricted to certain phylotypes, it would be interesting to evaluate the expression of DsA1 in atypical strains typed IA_2_ and II which were isolated from acne lesion. Indeed, due to the presence of repeat sequences towards the mid-region and carboxy-terminus as well as frameshifts disrupting the signal sequence, the sequence of DsA1 appears to be diverse which could generate DsA1 homologs proteins^31^. It would be necessary to elaborate tools able to identify DsA1 homologs and determined if such truncated proteins still have hFg-binding capacity.

*P. acnes* has never yet been described as invasive. However, it has been suggested that, in addition to direct cross-contamination during surgery, the presence of *P. acnes* in the oral compartment may account for some routes of infection in prosthetic infections, with the possible hematogenous spread of *P. acnes* to prosthetic joints after dental manipulations, dentogingival infections and periodontal disease^53^. Whether bacterial infection results from contamination or an invasive process, *P. acnes* needs to be able to colonize these niches. The DsA1/PA25597 gene is differentially expressed between *P. acnes* strains due to the presence of homopolymeric tracts (HPTs) in its sequence, providing *P. acnes* with adaptive capacity^16^. We suggest that DsA1 may be involved in this adaptation, like several other hFg-binding proteins from Gram-positive bacteria promoting bacterial survival in the host^54^. Indeed, hFg is also present in the ECM outside blood vessels, where it plays an important role in inflammation^55, 56^. Simultaneous proteomic analyses of pilosebaceous follicles from normal skin from the skin of patients with acne have shown that DsA1 is the most abundant protein produced by *P. acnes* and that hFg is one of the most strongly upregulated host proteins in the pilosebaceous follicle in acne patients^57^. Moreover, hFg seems to play an important role in *P. acnes* infection, because its concentration increases in a microenvironment mimicking that of acne^58^. The simultaneous presence of DsA1 and hFg in the pilosebaceous follicle may favor the formation of *P. acnes* clump as it is observed with *S. aureus* ClfA^+^, contributing to increase the inflammation reaction by confering phagocytosis resistance to *P. acnes* ^59^. Further investigations are currently underway, to improve our understanding of the relationship between *P. acnes* and hFg and the role of DsA1 in bacterial virulence.

## Materials and Methods

### Bacterial strains and growth conditions

*P. acnes* strain ATCC 6919 or NCTC737 [Type IA_1_] was obtained from the American Type Culture Collection (Manassas, VA) and was grown under anaerobic conditions, in reinforced clostridial liquid and solid medium (RCM) (Difco Laboratories, Detroit, MI). *P. acnes* was transferred from the bacterial stock in glycero onto RCM agar plates and incubated for five days under anaerobic conditions, with a GasPak™ EZ Anaerobic Container System (Becton Dickinson & Co, Sparks MD, USA). A single colony was transferred into 100 ml RCM and cultured in the same conditions. Glycerol was added at a final concentration of 15% to the bacterial suspension, which was then stored frozen at −80 °C. This stock suspension was referred to as the “start stock” and was used in all experiments. For routine culture, we used 100 ml RCM, and the bacteria were harvested after 5 days at 37 °C, by centrifugation at 7,000 × *g* for 10 min at 4 °C. Pellets were pooled and washed in about 30 ml of cold PBS and then centrifuged again, as described above. Finally, the bacterial pellet was suspended in PBS [1.5 mM KH_2_PO_4_, 2.7 mM Na_2_HPO_4_.7H_2_O, 0.15 M NaCl (pH 7.4)] (1:10 from volume culture). For large culture volumes, we used 200 ml of a five-day-old culture of *P. acnes* in RCM to inoculate 2 liters of RCM that had previously been equilibrated at 37 °C. Anaerobic conditions were maintained by flushing the culture thoroughly with N_2_ and then sealing it. At two-hour intervals, we harvested 10 ml of culture, to measure the absorbance at 620 nm and the pH, and the culture was flushed with N_2_, as described above. Bacteria were centrifuged at 7,000 × *g* for 10 min and the pellet was resuspended in PBS. The total surface protein extract was obtained as described below.

### Total surface protein extraction

Surface proteins were extracted by heating at 60 °C for 20 min in PBS alone or at 45 °C for 2 h in the presence of 1% LiCl^60^. Bacteria were removed by centrifugation at 16,000 × *g* for 20 min at 4 °C. Excess LiCl was removed by dialysis against PBS. The resulting solution, containing surface proteins, was concentrated by ammonium sulfate precipitation at 80% saturation for 18 h at 4 °C, with stirring^21^. The precipitated proteins were recovered after centrifugation at 15,000 × *g* for 30 min at 4 °C, then resuspended in PBS, and extensively dialyzed against PBS. Protein concentration was determined by the Lowry method, using BSA as the standard.

### Biotinylation

Concentrated surface protein extract and purified proteins were adjusted to a concentration of 10 mg/ml in PBS and dialyzed against [74 mM sodium tetraborate, 60 mM boric acid (pH 8.8)] overnight at 4 °C. Proteins were incubated with the extrinsic labeling reagent sulfo-N-hydroxysuccinimide (NHS)-biotin (Sigma) at a ratio of 250 μg NHS-biotin per 1 mg of protein for 4 h at 4 °C, with end-over-end rotation. The reaction was stopped by adding 1 M NH_4_Cl. Excess biotin-NHS was removed by dialysis against PBS at 4 °C. Biotinylated protein preparations were stored at −80 °C before use.

### Binding activity

We characterized the interaction between *P. acnes* and ligands, using biotinylated molecules in quantitative and qualitative assays. In a first set of assays, we used ECM ligands (human fibrinogen (hFg), collagens I, IV, VI and VIII) labeled with biotin. In a second set of assays, *P. acnes* surface proteins (total extract and purified DsA1) were biotinylated. *For quantitative analysis*, unlabeled protein was diluted with 50 mM carbonate-bicarbonate buffer (pH 9.6), to obtain a protein concentration of 0.8 to 50 μg/ml, and then immobilized on 96-well polystyrene plates by incubation overnight at 4 °C. The wells were washed three times with 0.2 ml of 0.05% Tween-20 in PBS (PBS-Tween). Biotinylated proteins (0.01 to 16 μg/ml in PBS-Tween) were added to the wells and the plates were incubated at room temperature for 1 h. The wells were washed three times with 0.2 ml PBS-Tween. Peroxidase-conjugated streptavidin (0.5 μg/ml in PBS-Tween) was added to the wells, and the plates were incubated for 30 min at room temperature. The plates were then washed, and bound peroxidase was detected by incubation with the chromogenic peroxidase substrate ABTS. *For qualitative analysis*, unlabeled proteins (10–50 μg per lane) were separated by SDS-PAGE in a 10% acrylamide gel and transferred to nitrocellulose membranes (pores: 0.45 μm diameter). Membranes were then saturated by incubation overnight at 4 °C with 5% BSA, 0.05% Tween-20 in PBS (PBT buffer). Binding activity was detected by incubating the membrane with 20 ml of a 0.1 μg/ml solution of biotinylated proteins in PBT for 2 h at room temperature, and then washing the membrane three times with PBT. Bound biotinylated proteins were detected by incubating the membrane with peroxidase-conjugated streptavidin (0.5 μg/ml in PBT) for 1 h at room temperature. The membrane was washed and bound peroxidase activity was detected by incubation with 3,3′-diaminobenzidine in the presence of CoCl_2_ and H_2_O_2_^61^.

### Two-dimensional electrophoresis

A 13 cm immobilized pH gradient (pH 10–3 IPG strip; GE Healthcare) was rehydrated by incubation at 20 °C for 13 h with 250 μl IEF solution (8 M urea-2% CHAPS (wt/vol)-0.5% IPG buffer pH 4–7 (vol/vol)-0.002% bromophenol blue) supplemented with 200 μg of protein from the concentrated surface protein extract. Isoelectric focusing was conducted at 20 °C in four steps, 1 h at 200 V, 1 h at 500 V, 30 min at 8000 V in gradient mode, and 3 h at 8000 V with the Ettan IPGphor system (VWR). For the second dimension, the IPG strip was equilibrated by incubation for 15 min in 6 M urea-30% glycerol (wt/vol)-0.05 M Tris-HCl-2% SDS (wt/vol)-0.002% bromophenol blue-100 mM DTT with gentle rocking, and then for 15 min in 6 M urea-30% glycerol (wt/vol)-0.05 M Tris-HCl-2% SDS (wt/vol)-0.002% bromophenol blue-400 mM iodoacetamide. The IPG strip was then applied onto a 12% acrylamide SDS-PAGE gel. Typically, two gels were run in parallel for 6 h at a constant current of 70 mA. One gel was subjected to silver staining without the glutaraldehyde step, for protein detection. The spot of interest was visualized in the binding assay, as previously described. The silver-stained gels and membranes were aligned and the spots of interest were excised from the gel.

### Peptide mass fingerprinting by MALDI-ToF mass spectrometry

In-gel tryptic digestion of two-dimensional protein spots was carried out with excised gel plugs (3.5 mm), which were washed in 50% acetonitrile (ACN) and 50 mM NH_4_CO_3_ (v/v). The plugs were then dried for 30 min, and the proteins they contained were digested in 25 μl ammonium bicarbonate buffer (pH 8.0) supplemented with 0.5 μg modified trypsin (Promega, Madison, WI, USA, sequencing grade) for 6 h in a thermomixer (Eppendorf) at 37 °C, with vortexing at 500 rpm. The supernatant of the tryptic digest and the peptides remaining in the gel were subjected to two rounds of extraction in 50% ACN with 5% trifluoric acid (TFA) (v/v). All liquids were pooled and dried and the peptides obtained were resuspended directly in 10 μl of 0.3% TFA. One microliter of sample was mixed on the stainless steel MALDI plate with 1 μl of 4 mg/ml CHCA (R-cyano-4-hydroxycinnamic acid, Sigma Aldrich) in ACN/0.3% TFA (50:50; v/v) and dried at room temperature. Mass spectra were acquired on a Voyager DE-STR+ time-of-flight mass spectrometer (Applied Biosystems) equipped with a 337-nm nitrogen laser. Spectra were recorded in positive reflector mode, with an accelerating voltage of 20 kV, a delayed extraction time of 130 ns, and a 62% grid voltage. In cases of high background noise, the CHCA matrix was replaced with 10 mg/ml DBH matrix (2,5-dihydroxybenzoic acid, Sigma Aldrich) in ACN/0.3% TFA (50:50; v/v). Ammonium phosphate (final concentration, 10 mM) was added to peptides of interest with peaks overlapping the matrix cluster peaks of CHCA. Spectra were obtained for the 700 to 4000 Da mass range, with internal calibration based on autolytic trypsin fragments characterized by (M + H)^+^  = 842.509 and 2211.104 Da. For protein identification, MS/MS peak lists were extracted, converted into mzdata.xml format files and compared with the protein database, using the MASCOT Daemon (version 2.1.3; Matrix Science, London, UK) search engine. The searches were performed with no fixed modification, with variable modifications for methionine oxidation, and with a maximum of one missed cleavage. Only peptides matching an individual ion score > 60 were considered. Proteins with two or more unique peptides matching the protein sequence were automatically considered to give a positive identification.

### Fibrinogen deglycosylation

We used endoglycosidases, N-glycanase and O-glycanase to remove glycans from hFg (Prozyme, San Leandro, CA, USA). Purified commercial human fibrinogen (hFg, 100 μg; Sigma) was denatured by heating at 100 °C for 5 min in a buffer containing 0.4% SDS, 200 mM β-mercaptoethanol, 50 mM sodium phosphate (pH 7.0). It was then cooled to room temperature and 3% NP-40 was added. For the removal of N-linked glycans, we added 0.5 U of recombinant *Flavobacterium meningosepticum* PNGase F (*N*-glycanase) produced in *Escherichia coli* and incubated the mixture for 24 h at 37 °C in a final volume of 50 μl. For the removal of O-linked glycans, denaturated hFg was first incubated in the presence of 0.25 U *Vibrio cholerae* sialidase A, bovine testis β-galactosidase, and jack bean meal β-N-acetyl-glucosaminidase for 3 h at 37 °C, to ensure the removal of monosaccharides such that only the Galβ(1–3)GalNAc core remained attached to the protein. We then added 0.5 U of recombinant *Streptococcus pneumoniae* Endo-α-N-acetyl-galactosaminidase (O-glycanase) produced in *Escherichia coli* and incubated the mixture for 24 h at 37 °C. Enzymatic reactions were stopped by mixing the sample with the electrophoresis sample buffer, and then denaturing by heating at 100 °C for 3 min. We then assessed the ability of N- and O-deglycosylated fibrinogen to recognize biotinylated DsA1, in the binding assay described above. The deglycosylation reaction was monitored by looking for mobility shifts for the h Fg bands following deglycosylation, on SDS-PAGE gels stained with Coomassie blue, and using RCA-I and jacalin plant lectin binding assays, as described below.

### Glycosyl composition of DsA1, as determined with alditol acetates

Concentrated surface protein extract was subjected to electrophoresis, and the resulting bands were transferred onto PVDF membrane, stained with Ponceau S stain, and the DsA1 band was excised (9 bands). The PVDF-bound protein was extracted as previously described^62^. Briefly, the membrane fragment carrying the PVDF-bound protein band was cut into several pieces, which were placed in a plastic tube. The extraction solvent (500 μl), 70% acetonitrile and 1% TFA, was added and the tube was vortexed for 5 min. The sample was then subjected to sonication for 30 min at room temperature and incubated at 42 °C overnight. It was then subjected to sonication for a further 30 min. The solution was recovered and the PVDF pieces were rinsed with 200 μl fresh extraction solvent. The protein-containing solutions were pooled and dried under a stream of nitrogen, and proteins were then hydrolyzed in 300 μl of 4 M TFA at 100 °C for 4 h. The TFA was removed by drying under a vacuum, and sugar residues were reduced with 200 μl NaBH_4_ (10 mg/ml in 2 M NH_4_OH). The resulting alditols were per-*O*-acetylated and analyzed by gas chromatography, as previously described^63^.

### Plant lectin binding activity

Proteins (50 μg) were subjected to SDS-PAGE in a 10% acrylamide gel, and the resulting bands were transferred onto nitrocellulose membranes. The membrane was blocked by incubation with [0.5% BSA, 0.15 M NaCl, 0.1 mM CaCl_2_, 0.01 mM MnCl_2_.4H_2_O, 0.01 M HEPES.Na^+^ (pH 7.5)] (HEPES-BSA) overnight at 4 °C, and plant lectin-binding activity was detected by incubating the membrane with HEPES-BSA containing biotinylated ConA, SBA, PNA, SNA, MAL-II, RCA-I, jacalin, UEA-I, DBA, and WGA plant lectins (0.2 μg/ml; Vector Laboratories, Inc., Burlingame, CA, USA) (See Table 2 for binding specificities) for 1 h at room temperature. The membrane was then washed three times, for 15 min each, with HEPES-BSA. Bound biotinylated lectin was detected by incubating the membranes with peroxidase-conjugated streptavidin (0.5 μg/ml) for 30 min at room temperature. The membranes were washed three times, for 15 min each, with HEPES-BSA, and bound peroxidase activity was detected by incubation with 3,3′-diaminobenzidine in the presence of CoCl_2_ and H_2_O_2_^61^.

### Production and purification of the GST-fusion proteins

The *E. coli* BL21DE3pLys strain was used to produce the GST-fibrinogen-fragment fusion proteins (Supplementary Figs S2 and S3). Bacteria were grown overnight in 10 ml LB medium supplemented with 100 μg/ml ampicillin and 40 μg/ml chloramphenicol, and were used to inoculate 1 liter of LB medium. When the culture, incubated at 30 °C, reached an OD of 0.7 at 620 nm, protein production was induced by adding 0.5 mM isopropyl β-D-1-thiogalactopyranoside (IPTG), and culture was continued for another four hours. Bacteria were harvested by centrifugation at 5,000 × *g* for 10 min and the pellet was washed once in PBS and resuspended in TEN-T lysis buffer (20 mM Tris-HCl pH 7.5, 0.5 mM EDTA, 150 mM NaCl, 1% Triton X-100). The suspension was subjected to pulses of sonication for 30 seconds on ice, and was then supplemented with 2 mM DTT, 1.5% N-lauryl sarcosine. The lysate was centrifuged for 20 min at 20,000 × *g* and the pellet was discarded. The supernatant was loaded onto a glutathione Sepharose 4B column (GE Healthcare) and the purified protein was eluted in 50 mM Tris, 10 mM glutathione, pH 8.0. Fractions containing the recombinant peptide (as shown by SDS-PAGE) were pooled. In a final step, just before use, the supernatant was dialyzed against PBS to remove N-lauryl sarcosine.

### Statistical analysis

The statistical significance of differences between data from experimental groups was analyzed by paired Student’s-test. A level of P < 0.05 was accepted as significant.

## Electronic supplementary material

Supplementary Information
