# Supplementary material for: Characterization of a Propionibacterium acnes Surface Protein as a Fibrinogen-Binding Protein
Source: Sci Rep. 2017 Jul 25;7:6428. doi: 10.1038/s41598-017-06940-3 (PMC5527093; doi:10.1038/s41598-017-06940-3)
Supplement: Supplementary file 1 — Supplementary Information [file 41598_2017_6940_MOESM1_ESM.pdf]

Revised Manuscript SREP-17-12267 Version 1

Characterization of a *Propionibacterium acnes* Surface Protein as a Fibrinogen-Binding Protein

Philippe A. Grange[1], Joël Raingeaud[2], Willy Morelle[3], Anne-Geneviève Marcelin[4], Vincent Calvez[4], Nicolas Dupin[1,5]*

[1]Université Sorbonne Paris Descartes, Faculté de Médecine, INSERM CNRS UMR8104, Institut Cochin U1016, Laboratoire de Dermatologie-CNR Syphilis, Paris, France. [2]INSERM U981, Institut Gustave Roussy, Villejuif, France. [3]UMR CNRS/USTL 8576, Unité de Glycobiologie Structurale et Fonctionnelle, Université des Science et Technologies de Lille 1, France. [4] AP-HP, Groupe Hospitalier Pitié-Salpêtrière – Charles Foix, Service de Virologie – INSERM 1136-UMR UPMC Paris 6, Paris, France. [5]AP-HP, Groupe Hospitalier Paris Centre Cochin-Hôtel Dieu-Broca, Service de Dermatologie-Vénéréologie, Paris, France.

SUPPLEMENTARY MATERIAL

Purification of DsA1. Concentrated surface protein extract (90 mg of protein in 16 ml) was centrifuged at 5,000 x *g* for 10 min at room temperature to remove unsolubilized material, and was then fractionated by anion-exchange chromatography on a UNOsphere Q anion exchange column (2.5 by 10 cm) (BioRad) equilibrated in buffer A [25 mM Tris (pH 8.0)], at a flow rate of 24 ml/h. Unbound proteins were washed with buffer A (24 ml), and bound proteins were eluted stepwise with a 0 to 160 mM (28 ml) buffer A, 160 to 200 mM (40 ml) buffer A + 2 M NaCl gradient. The fractions (1.5 ml) containing hFg-binding activity were pooled, desalted by extensive dialysis at 4°C against 0.1 M NH_4HCO_3, pH 8.0, and

freeze-dried. Final purification of the DsA1 protein was achieved by loading 3 mg of protein in a volume of 5 ml onto a Sephacryl S-300 HR column (1 by 120 cm) (GE Healthcare) equilibrated with 0.1 M NH_4HCO_3, pH 8.0, and eluting at a flow rate of 6 ml/h (1.5 ml/fraction) over a period of 24 h. The fractions (1.5 ml) containing hFg-binding activity were pooled and stored at -20°C.

Table S1: Purification of DsA1.

Purification step	Protein (mg)	Total units (U)	Specific Activity (U/mg)	DsA1 recovery (%)
Crude lithium extract	1343	390000	290	/
Salt precipitation	378	160000	423	100
Anion exchange	2.9	3000	1034	1.87
Gel filtration	0.2	368	1840	0.23

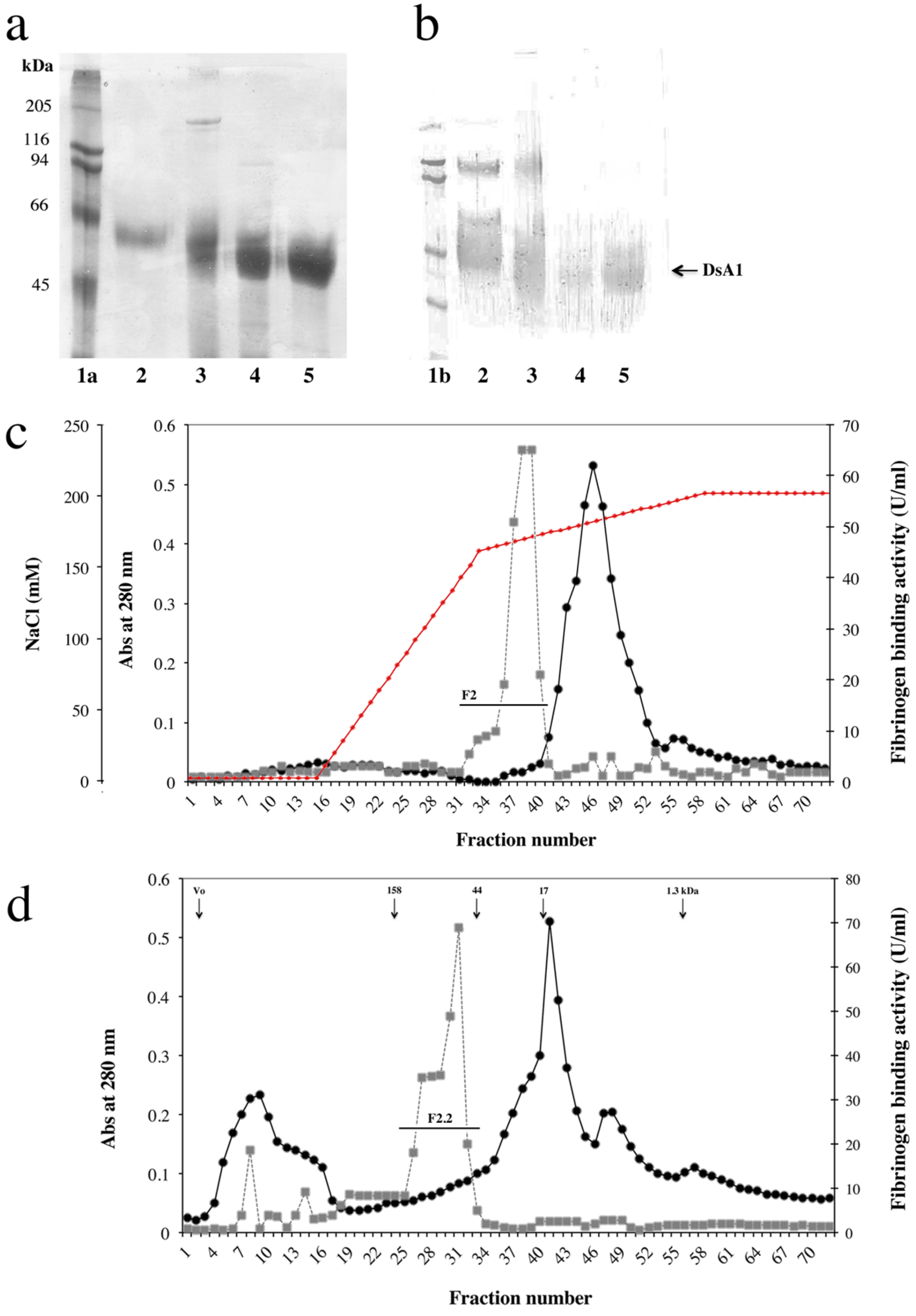
a
kDa
205
116
94
66
45
1a
2
3
4
5
b
1b
2
3
4
5
← DsA1
c
NaCl (mM)
250
200
150
100
50
0
Abs at 280 nm
0.6
0.5
0.4
0.3
0.2
0.1
0
Fibrinogen binding activity (U/ml)
70
60
50
40
30
20
10
0
F2
1 4 7 10 13 16 19 22 25 28 31 34 37 40 43 46 49 52 55 58 61 64 67 70
Fraction number
d
Abs at 280 nm
0.6
0.5
0.4
0.3
0.2
0.1
0
Fibrinogen binding activity (U/ml)
80
70
60
50
40
30
20
10
0
Vo
158
44
17
1.3 kDa
F2.2
1 4 7 10 13 16 19 22 25 28 31 34 37 40 43 46 49 52 55 58 61 64 67 70
Fraction number

Figure S1: Purification of DsA1. (**a**) Proteins were separated by 10% SDS-PAGE stained with Coomassie blue, and (**b**) detected with biotinylated hFg. Lanes 1a/b contain unlabeled and biotinylated molecular weight standards, respectively. Lane 2: Lithium chloride *P. acnes* total surface protein extract (10 μg). Lane 3: concentrated surface protein (10 μg). Lanes 4 and 5: 10 μg of fractions F2 and F2.2, respectively. (**c**) Concentrated surface protein (85 mg) was loaded (24 ml/h) onto an UNOsphere Q column equilibrated with 25 mM Tris, pH 8.0. Proteins were eluted with linear gradients of 0 to 160 mM NaCl in 25 mM Tris pH 8.0 for 60 min and 160 to 200 mM NaCl in 25 mM Tris, pH 8.0 for 90 min. Pooled fractions containing DsA1 were desalted and equilibrated with 0.1 M NH_4HCO_3, pH 8.0. (**d**) Proteins were fractionated on a Sephacryl HR S300 column, at 6 ml/h. Void volume (Vo) was determined with thyroglobulin (669 kDa), and the elution positions for bovine γ-globulin (158 kDa), chicken ovalbumin (44 kDa), equine myoglobin (17 kDa) and vitamin B12 (1.3 kDa) are indicated by arrows. Protein concentration was monitored at 280 nm (●). Fibrinogen-binding activity (■) was determined with biotinylated hFg. Horizontal lines labeled F2 and F2.2 indicate the pooled fractions containing hFg-binding activity.

Plasmid contructs. Fragments of the Bβ-subunit of hFg (GenBank Accession number NG008833.1) were obtained by RT-PCR from total RNA extracted from Hep-G2 human hepatoma cells cultured in modified Dulbecco's medium supplemented with 10% fetal calf serum. Briefly, total RNA was isolated with TRIzol reagent (Invitrogen Ltd, Paisley, UK) according to the manufacturer's instructions and treated with DNAse I (Roche Molecular Biochemicals). RNA concentration was determined by measuring absorbance at 260 nm (NanoDrop). Complementary DNA was generated from 2 μg total RNA with an oligo(dT) primer and 200 U of SuperScript™ II Reverse Transcriptase (Invitrogen Ltd., Paisley, UK), and was then used as the template for standard PCR. Standard amplification was carried out with 0.5 U of high-fidelity Platinum® *Pfx* DNA polymerase (Invitrogen) in a final volume of 25 μl and the following cycling conditions:35 cycles of 94°C for 15 s, 50°C for 30 s and 68°C for 45 s. The specific pairs of primers used were: for Fg1: GCAGGAATTCTGATGAAAAGGATGGTTTCTTGG, GGCCGCTCGAGTACACAACACCCCCAGGTCTGG; Fg2: GCAGGAATTCTGGATGCTGGAGGCTGTCTTCAC, GGCCGCTCGACTAGACACCACAGGAATATTGCA; Fg3: GCAGGAATTCTGAAGTT AGAATCTGATGTCTCA, GGCCGCTCGAGTTTCATTCTGTACAGTGAATCC; Fg4: GCAGGAATTCTGCCCACAGAACTTTTGATAGAA, GGCCGCTCGAGTCTGTGGGA AGAAGGGCCTGAT; amplifiying 318, 441, 462 and 462 bp fragments, respectively. We added restriction sites for *Eco*RI and *Xho*I to the sense- and antisense primers, respectively. Fibrinogen PCR fragments were inserted into the pBluescript-SK$^+$ vector and sequenced to confirm integrity. They were then inserted into the pGEX-4T-2 plasmid digested with *Eco*RI and *Xho*I.

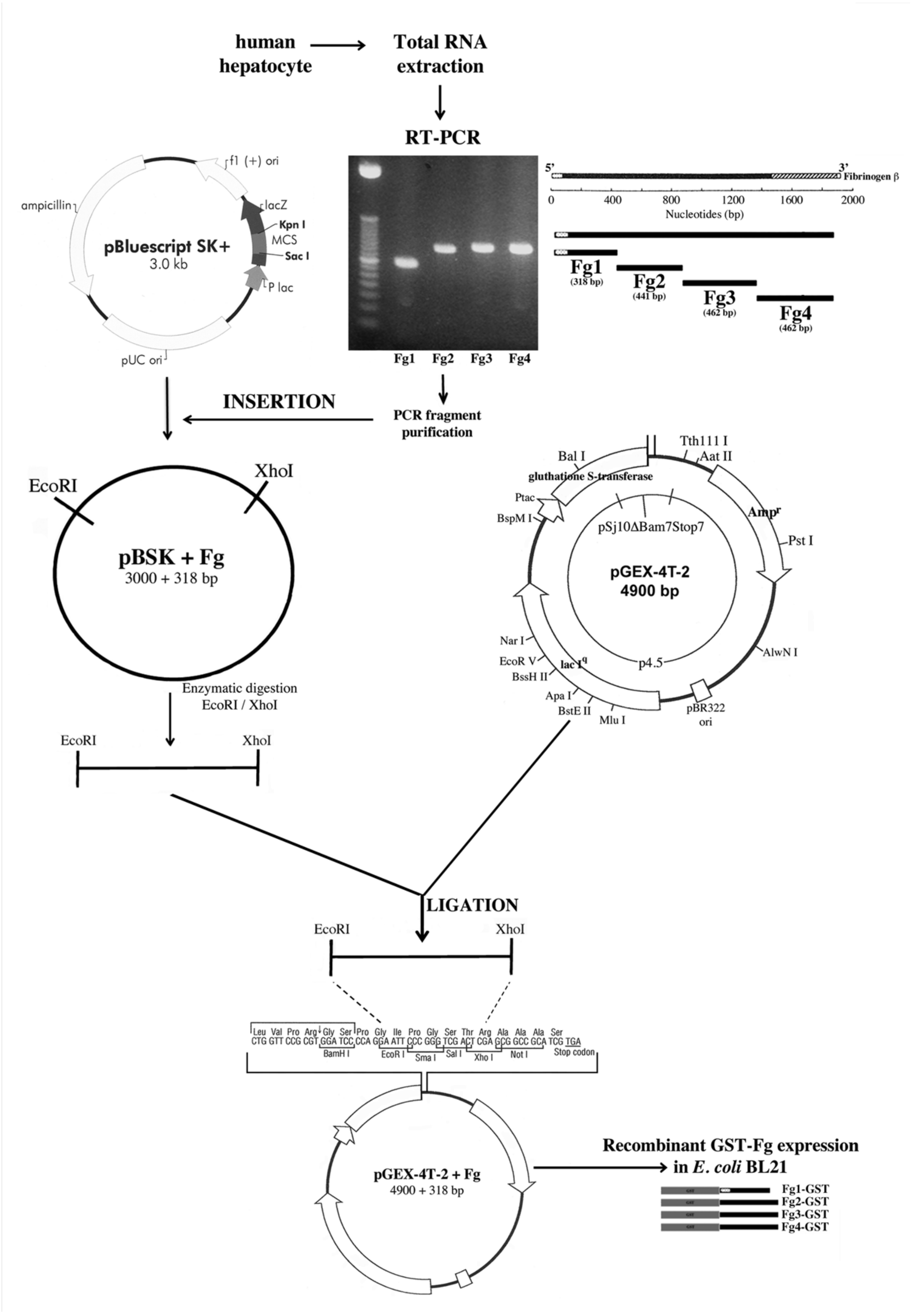

Figure S2: Cloning of Bβ fragments of human fibrinogen. Constructs and expression of recombinant human fibrinogen Bβ fragments Fg1, Fg2, Fg3 and Fg4. Fibrinogen fragments were obtained by RT-PCR and expression plamids were contructed.

1 10 20 30 40 50

MKRMVSWSFH KLKTMKHLLL LLLCVFLVKS QGVNDNEEGF FSARGHRPLD

KKREEAPSLR PAPPPISGGG YRARPAKAAA TQKKVERKAP DAGGCLHADP

DLGVLCPTGC QLQEALLQQE RPIRNSVDEL NNNVEAVSQT SSSSFQYMYL

LKDLWQKRQK QVKDNENVVN EYSSELEKHQ LYIDETVNSN IPTNLRVLRS

ILENLRSKIQ KLESDVSAQM EYCRTPCTVS CNIPVVSGKE CEEIIRKGGE

TSEMYLIQPD SSVKPYRVYC DMNTENGGWT VIQNRQDGSV DFGRKWDPYK

QGFGNVATNT DGKNYCGLPG EYWLGNDKIS QLTRMGPTEL LIEMEDWKGD

KVKAHYGGFT VQNEANKYQI SVNKYRGTAG NALMDGASQL MGENRTMTIH

NGMFFSTYDR DNDGWLTSDP RKQCSKEDGG GWWYNRCHAA NPNGRYYWGG

QYTWDMAKHG TDDGVVWMNW KGSWYSMRKM SMKIRPFFPQ Q

Figure S3: Amino-acid sequence of peptide fragments generated from human fibrinogen. Peptide fragments Fg1 (37 kDa) (bold), Fg2 (43 kDa) (underlined), Fg3 (43 kDa) (highlighted in gray), and Fg4 (43 kDa) (double-underlined) were generated from the Bβ-subunit of human fibrinogen (GenBank accession number NG008833.1).

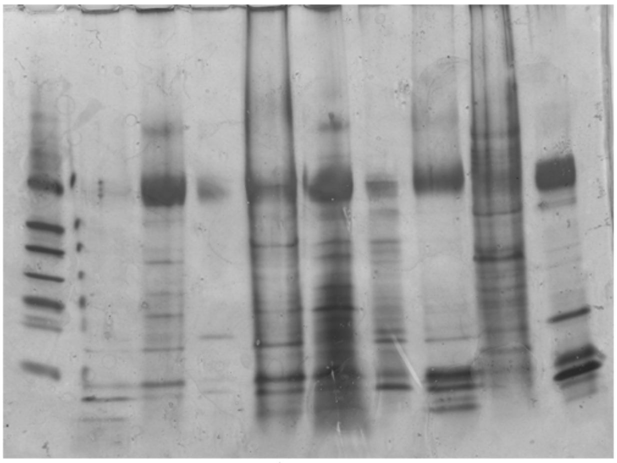

1 2 3 4 5 6 7 8 9 10

Figure S4 : Supplemental to figure 1a. Silver staining of *P. acnes* surface proteins extracts (10 µg per lane) electrophoretically separated. Lanes 6 and 8 correspond to lanes 3 and 2 in figure 1a. Lane 1 : from up to down, molecular weigh standards correspond to 66, 45, 36, 29, 24, 20 and 14.2 kDa.

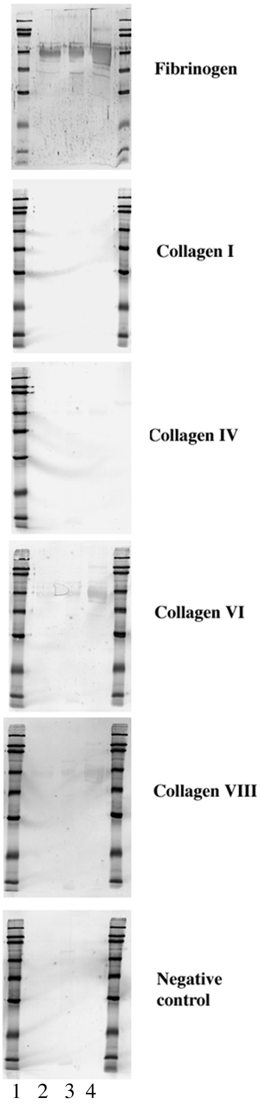

Figure S5 : Supplemental to figure 1b. *P. acnes* surface protein extracts were electrophoretically separated (50 μg per lane), transferred onto nitrocellulose membrane and incubated with biotinylated human fibrinogen, collagens I, IV, VI and VIII (Fg, ColI, ColIV, Col VI, Col VIII; 0.1 μg/ml). Negative control was performed with HRP-streptavidin alone. Lanes 2 to 4 : surface proteins extracted at 60°C in PBS, at 45°C in 1 M LiCl, and at 60°C in presence of 2% SDS, respectively. Lane 1: from up to down, biotinylated molecular weigh standards correspond to 180, 116, 97, 58, 39, 29, 20, and 14 kDa. Lanes 2 and 3 correspond to lanes 3 and 2 in figure 1b, respectively.

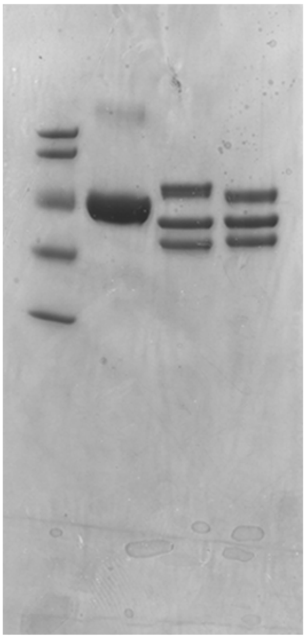

Figure S6: Supplemental to figure 4b. Commassie blue staining of proteins (10 μg per lane) electrophoretically separated. Lanes 2 to 4: BSA, hFg, bFg respectively. Lane 1 : from up to down, molecular weigh standards correspond to 116, 97, 66, 45 and 29 kDa. Lanes 2 and 3 correspond to lanes 2 and 3 in figure 1b, respectively.

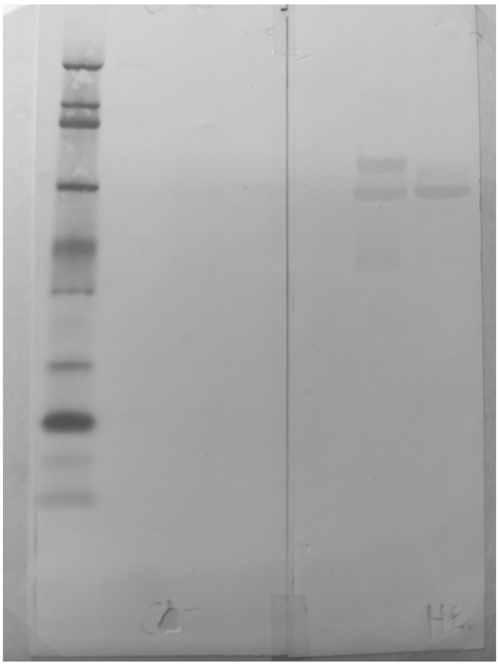

1 2 3 4 2 3 4

Figure S7: Supplemental to figure 4c, d. Proteins were electrophoretically separated (50 µg per lane) and transferred onto nitrocellulose membrane. Left panel: negative control HRP-streptavidin alone. Right panel : binding activity with biotinylated DsA1 (0.1 µg/ml). Lanes 2 to 4 : BSA, hFg, bFg respectively. From up to down, biotinylated molecular weigh standards correspond to 180, 116, 97, 58, 39, 29, 20, and 14 kDa. Lanes 2 and 3 correspond to lanes 2 and 3 in figures 4c and 4d, respectively.

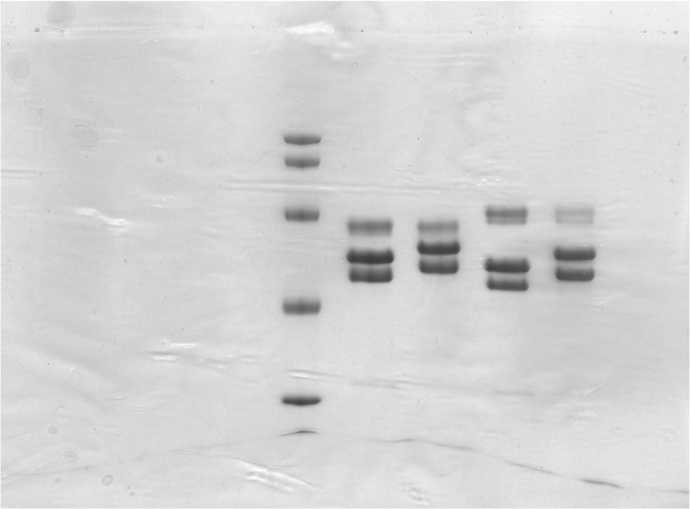

Figure S8: Supplemental to figure 4e. Commassie blue staining of human and bovine fibrinogen (10 μg per lane) electrophoretically separated. Lanes 2 and 3: hFg. Lanes 4 and 5: bFg. Lanes 2 and 4 : N-glycosidase F (PNGAse F) treated; lanes 3 and 5 : untreated. Lane 1 : from up to down, molecular weigh standards correspond to 116, 97, 66, 45 and 29 kDa. Lanes 2 and 3 correspond to lanes 5 and 4 in figure 4e, respectively.

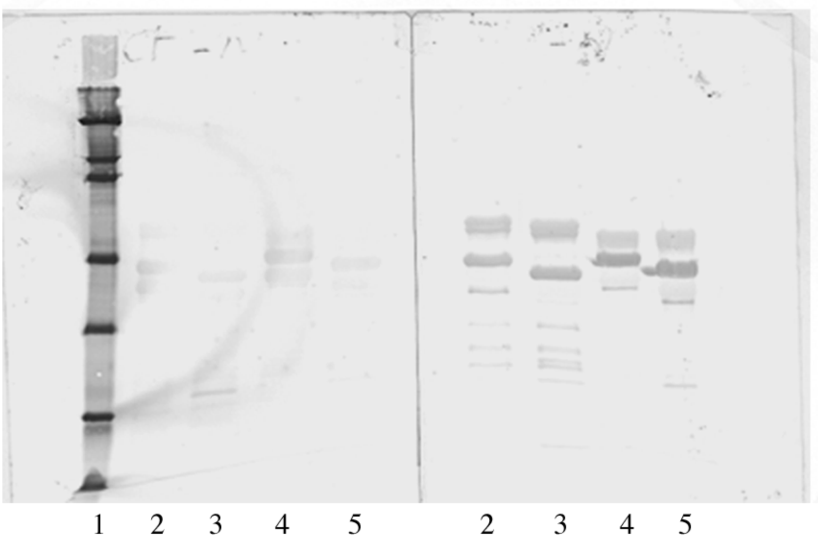

Figure S9: Supplemental to figure 4f. Human and bovine fibrinogen were electrophoretically separated (50 µg per lane), transferred onto nitrocellulose membrane and incubated with biotinylated DsA1 (0.1 µg/ml). Lanes 2 and 3: hFg. Lanes 4 and 5: bFg. Lanes 2 and 4 : untreated; lanes 3 and 5 : N-glycosidase F (PNGAse F) treated. Lane 1: from up to down, biotinylated molecular weigh standards correspond to 180, 116, 97, 58, 39, 29, 20, and 14 kDa. Left panel: negative control HRP-streptavidin alone. Right panel : Binding activity with biotinylated DsA1 (0.1 µg/ml). Lanes 2 and 3 correspond to lanes 4 and 5 in figure 4f, respectively.

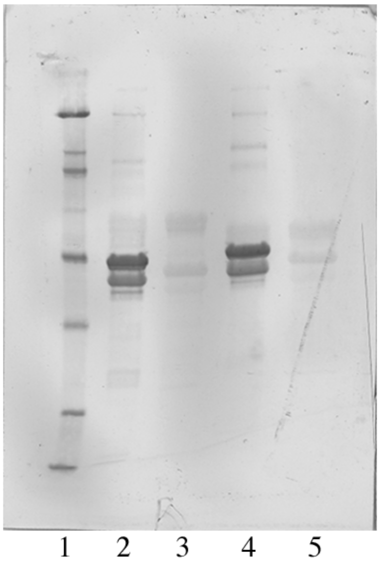

Figure S10: Supplemental to figure 4g. Human and bovine fibrinogen were electrophoretically separated (50 μg per lane), transferred onto nitrocellulose membrane and incubated with biotinylated RCA-1 lectin. Lanes 2 and 3: hFg. Lanes 4 and 5: bFg. Lanes 2 and 4 : untreated. Lanes 3 and 5 : N-glycosidase F (PNGAse F) treated. Lane 1: from up to down, biotinylated molecular weigh standards correspond to 180, 116, 97, 58, 39, 29, 20, and 14 kDa. Left panel: negative control HRP-streptavidin alone. Right panel : Binding activity with biotinylated DsA1 (0.1 μg/ml). Lanes 2 and 3 correspond to lanes 4 and 5 in figure 4g, respectively.

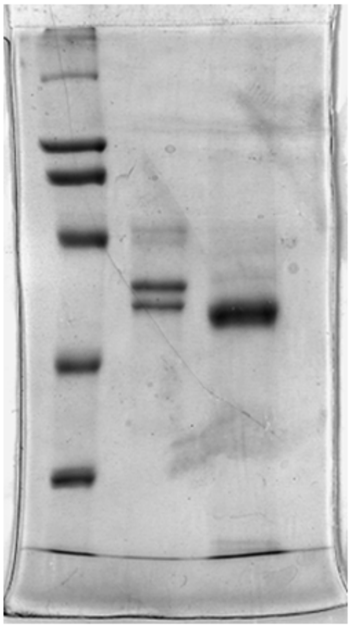

1 2 3

Figure S11: Supplemental to figure 4h. Commassie blue staining of human fibrinogen (10 µg per lane) electrophoretically separated. Lanes 2 and 3 : untreated and O-glycosidase treated, respectively. Lane 1 : from up to down, molecular weigh standards correspond to 116, 97, 66, 45 and 29 kDa. Lanes 2 and 3 correspond to lanes 4 and 6 in figure 4f, respectively.

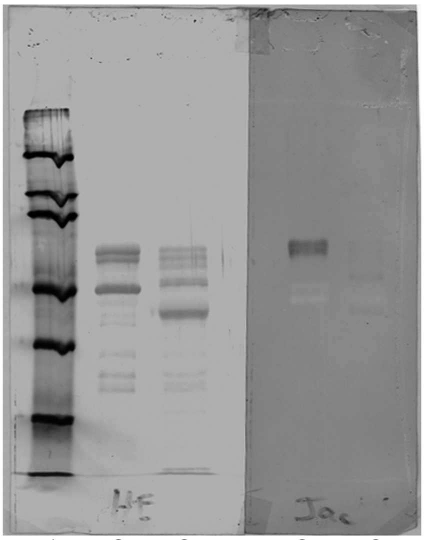

1 2 3 2 3

Figure S12: Supplemental to figure 4i and 4j. Human fibrinogen was electrophoretically separated (50 μg per lane), transferred onto nitrocellulose membrane and incubated with biotinylated jacalin lectin. Lanes 2 and 3: untreated and O-glycosidase treated, respectively. Lane 1 : from up to down, molecular weigh standards correspond to 116, 97, 66, 45 and 29 kDa. Lanes 2 and 3 correspond to lanes 4 and 6 in figures 4i and 4j, respectively.
